# Synergistic effects of retinol and retinyl palmitate in alleviating UVB-induced DNA damage and promoting the homologous recombination repair in keratinocytes

**DOI:** 10.3389/fphar.2025.1562244

**Published:** 2025-04-24

**Authors:** Jiangming Zhong, Ling Liang, Nan Zhao, Jing Wang, Peng Shu

**Affiliations:** HBN Research Institute and Biological Laboratory, Shenzhen Hujia Technology Co., Ltd., Shenzhen, Guangdong, China

**Keywords:** retinol, retinyl palmitate, DNA damage, UVB, homologous recombination

## Abstract

**Background:**

Ultraviolet B (UVB) rays are a type of ultraviolet radiation emitted by the sun, primarily responsible for skin photodamage. These rays mainly affect the epidermis, leading to direct damage to DNA and contributing to skin cancer development. Retinol and its derivatives are effective in combating skin aging and photodamage, but they often cause skin intolerance, limiting their use despite their potent effects. Therefore, investigating optimal compositions of retinoids is essential to enhance their efficacy against photodamage.

**Method:**

In this study, we investigated the synergistic effects of retinol (ROL) and retinyl palmitate (RPalm) in alleviating UVB-induced DNA damage in human keratinocytes (HaCaT) and reconstructed human epidermis. The ROL+RPalm combination was applied after UVB exposure. We utilized bulk mRNA sequencing, comet assays, Western blotting, immunofluorescence, and flow cytometry to evaluate the level of DNA damage and repair.

**Result:**

The application of the ROL+RPalm combination significantly reduced inflammation and apoptosis while promoting collagen synthesis compared to individual treatments with ROL or RPalm. Our findings indicated that the ROL+RPalm synergy primarily mediates DNA damage repair. Additionally, we elucidated that the molecular mechanism involves the activation of RARβ, which triggers the ATM-CHK2-p53 signaling pathway and increases the expression of homologous recombination (HR)-associated repair genes.

**Conclusion:**

This combination of ROL and RPalm presents a potential therapeutic strategy for UVB-induced photodamage and emphasizes the synergistic effects in alleviating UVB-induced DNA damage.

## 1 Introduction

Ultraviolet (UV) radiation from the sun is divided into three main types: UVC(100–290 nm), UVB (290–320 nm) and UVA (320–400 nm), with visible light having longer wavelengths. UV radiation can cause direct harm to biological systems or lead to damage indirectly by generating reactive oxygen species (ROS). This oxidative damage can result in inflammation, gene mutations, and photo-immunosuppression, contributing to the development of skin cancer (Halliday, 2005). Approximately 95% of the UV radiation that reaches the Earth’s surface consists of UVA, while UVB comprises about 5%. The ozone layer largely blocks UVC, so it seldom reaches human skin. Despite its lower prevalence, UVB is absorbed by DNA much more efficiently, with a tenfold difference in absorption between the two wavelengths (Budden and Bowden, 2013). Especially, UVB radiation is more potent than UVA in causing DNA damage, inducing higher injury at 200–800 mJ/cm^2^ within 4 h, compared to UVA’s 10–20 J/cm^2^, which causes single-strand breaks only after 24 h (Svobodova et al., 2012). Consequently, UVB can inflict significant damage at lower exposure levels, making it a major factor in skin photoaging and cancer.

The primary mechanism of UVB-induced DNA damage involves the formation of cyclobutane pyrimidine dimers (CPDs) and 6-4 photoproducts, which occur due to the abnormal linking of adjacent pyrimidine bases (Volatier et al., 2023). If these lesions are not adequately repaired, they can lead to mutations by affecting DNA replication and transcription (Hung et al., 2020; Walmacq et al., 2012). In response to UVB damage, several cellular pathways are activated, resulting in cell cycle arrest, apoptosis, or cellular senescence, depending on the severity of the injury (Yarosh et al., 2002). Therefore, protective measures such as antioxidants and DNA repair enzymes may offer strategies to combat the harmful effects of UVB radiation.

Retinoids, which are derivatives of vitamin A, have been extensively studied for their anti-aging properties in skin care products. Topical application of retinoids was found to decrease apoptotic cells in hairless mouse epidermis upon UVB exposure (Sorg et al., 2005). Retinol (ROL) is particularly noted for promoting collagen balance in the dermis by enhancing collagen production and inhibiting the expression of matrix metalloproteinases (MMPs) (Romana‐Souza et al., 2019). Additionally, ROL can help restore epidermal thickness (Li et al., 2017). Stabilized ROL (0.1%) has been shown to significantly improve photodamaged skin over long-term treatment. However, ROL is chemically unstable and prone to light and oxidation, limiting its effectiveness. To address this, retinol derivatives have been developed to enhance stability (Zhong et al., 2024). Among these, retinyl palmitate (RPalm) is more heat-stable and less irritating to the skin. In addition to boosting skin metabolism and collagen synthesis, RPalm effectively mitigates UVB-induced photoaging in both *in vitro* and *in vivo* (Shu et al., 2023). Compared to ROL, RPalm has also demonstrated superior ability to reduce inflammatory cytokines following UVB exposure (Shu et al., 2024).

Given these benefits, combining ROL and RPalm in anti-aging formulations is promising. However, the molecular mechanisms behind their synergistic effects remain unclear. This study aims to investigate the protective properties of ROL and RPalm against UVB-induced damage in human keratinocyte HaCaT cells. We identified an optimal concentration of ROL to RPalm and explored their efficacy in promoting DNA repair following UVB exposure.

## 2 Material and method

### 2.1 Cell culture and UVB irradiation

A spontaneously immortalized keratinocyte cell line, HaCaT, is derived from the adult human skin of a 62-year-old man. HaCaT cells were obtained from EK-Bioscience, China (CC-H1149). The cells were maintained in Dulbecco’s Modified Eagle’s Medium (DMEM, Gibco) (contain with 4.5 g/L D-Glucose, L- Glutamine, Phenol Red, and 110 mg/L Sodium Pyruvate) supplemented with 10% (v/v) fetal bovine serum (FBS, Gibco) and 1% (v/v) penicillin-streptomycin (P/S, Gibco). Cultures were incubated at 37°C in a humidified atmosphere containing 5% CO_2_. HaCaT cells were subjected to UVB irradiation using a UVB crosslinker (UCL-3500M, Luyor) with the cells covered by culture medium during irradiation. The actual dosage applied was 50 mJ/cm^2^, measured by a UV radiometer (Speedre, China, SDR-297) under culture medium. ROL (COACHCHEM, China) and RPalm (DSM-firmenich, the Kingdom of Netherlands) were used in this study. Both ROL and RPalm are in powder form and were dissolved in Dimethyl Sulfoxide (DMSO). The concentrations used in the experiment were 15 µM for ROL and 30 µM for RPalm.

### 2.2 Cell viability assay

Cell viability was assessed using the Cell Counting Kit-8 (CCK-8) (Beyotime, C0043) according to the manufacturer’s instructions. HaCaT cells (1 × 10^4^) were seeded in 96-well plates covered with 100 µL of culture medium overnight and then treated under 100 µL of specified conditions for 24 h. After treatment, the culture medium was discarded and replaced with 100 µL of CCK-8 solution per well, followed by incubation for 2 h at 37°C. The optical density (OD) of each well was measured at 450 nm using a microplate reader (Tecan Infinite E PLEX R, Switzerland) to evaluate cell viability. The optical density (OD) of the CCK-8 solution served as the blank control. The cellular viability for each treatment cohort was quantified using the formula: (OD value of the experimental group - OD value of the blank group)/(mean OD value of the control group - OD value of the blank group) * 100%. The number of technical and biological replicates was set to six.

### 2.3 Total RNA extraction and quantitative PCR (qPCR) analysis

Total RNA was extracted from the cells using the TransZol Up Plus RNA Kit (Transgen). The quantification of total RNA and determination of RNA integrity were performed utilizing Thermo Scientific NanoDrop spectrophotometer, ensuring A260/A280 absorbance ratios fell within the range of 1.8–2.1, and A230/A260 ratios were maintained between 2.0 and 2.2. 4 μg total RNA was reverse-transcribed into cDNA combined with 5X Evo M-MLV RT Reaction Mix (Agbio, AG11728) in a 20 µL reaction volume. Quantitative PCR analysis was performed by QuantStudio™ 1 Plus (Thermo Fisher) using a SYBR Green Mix (Yeasen, 11202 ES). The thermal cycling conditions were as follows: an initial denaturation step at 95°C for 5 min, followed by 40 cycles of denaturation at 95°C for 10 s and annealing/extension at 60°C for 60 s. Post-amplification, a melting curve analysis was conducted to validate the specificity of the amplification products. β-Actin served as the internal reference for quantifying gene expression levels. The ^2^(−ΔΔCt) method was employed to analyze the mRNA expression levels of the target genes. The sequences of the primers used are detailed in Table 1. All primer sequences were sourced from PrimerBank (PrimerBank).

**TABLE 1:** Primer sequences used for quantification of gene expression.

Gene	Forward primer (5′→3′)	Reverse primer (5′→3′)	Amplicon size
β-Actin	CAT​GTA​CGT​TGC​TAT​CCA​GGC	CTC​CTT​AAT​GTC​ACG​CAC​GAT	250
IL-6	ACT​CAC​CTC​TTC​AGA​ACG​AAT​TG	CCA​TCT​TTG​GAA​GGT​TCA​GGT​TG	149
IL-1β	ATG​ATG​GCT​TAT​TAC​AGT​GGC​AA	GTC​GGA​GAT​TCG​TAG​CTG​GA	132
TNF-α	CCT​CTC​TCT​AAT​CAG​CCC​TCT​G	GAG​GAC​CTG​GGA​GTA​GAT​GAG	220
HAS1	GAG​CCT​CTT​CGC​GTA​CCT​G	CCT​CCT​GGT​AGG​CGG​AGA​T	110
HAS2	CTC​TTT​TGG​ACT​GTA​TGG​TGC​C	AGG​GTA​GGT​TAG​CCT​TTT​CAC​A	205
HAS3	CAG​CCT​ATG​TGA​CGG​GCT​AC	CCT​CCT​GGT​ATG​CGG​CAA​T	210
CD44	CTG​CCG​CTT​TGC​AGG​TGT​A	CAT​TGT​GGG​CAA​GGT​GCT​ATT	109
MSH2	AGG​CAT​CCA​AGG​AGA​ATG​ATT​G	GGA​ATC​CAC​ATA​CCC​AAC​TCC​AA	176
MSH3	GTG​GCA​AAA​GGA​TAT​AAG​GTG​GG	AAA​GGG​CAG​TCA​ATT​TCC​GGG	109
LIG4	AGC​AAA​AGT​GGC​TTA​TAC​GGA​TG	TGA​GTC​CTA​CAG​AAG​GAT​CAT​GC	162
OGG1	ATG​GGG​CAT​CGT​ACT​CTA​GC	CTC​CCT​CCA​CCG​GAA​AGA​T	117
XPA	CCA​GGA​CCT​GTT​ATG​GAA​TTT​GA	GCT​TCT​TGA​CTA​CCC​CAA​ACT​TC	317
XPD	GGA​AGA​CAG​TAT​CCC​TGT​TGG​C	CAA​TCT​CTG​GCA​CAG​TTC​TTG​A	102
XRCC6	GTT​GAT​GCC​TCC​AAG​GCT​ATG	CCC​CTT​AAA​CTG​GTC​AAG​CTC​TA	249
XPF	GGA​ACT​GCT​CGA​CAC​TGA​CG	GCG​AGG​GAG​GTG​TTC​AAC​TC	187
XPC	CTT​CGG​AGG​GCG​ATG​AAA​C	TTG​AGA​GGT​AGT​AGG​TGT​CCA​C	199
ERCC1	CCT​TAT​TCC​GAT​CTA​CAC​AGA​GC	TAT​TCG​GCG​TAG​GTC​TGA​GGG	76
LIG3	TCA​CTG​GCG​TGA​TGT​AAG​ACA	CCT​GGA​ATG​ATA​GAA​CAG​GCT​TT	101
BRCA1	GAA​ACC​GTG​CCA​AAA​GAC​TTC	CCA​AGG​TTA​GAG​AGT​TGG​ACA​C	88
BRCA2	CAC​CCA​CCC​TTA​GTT​CTA​CTG​T	CCA​ATG​TGG​TCT​TTG​CAG​CTA​T	250
RAD54	GCT​GAG​CCC​ATG​AGT​GAA​AG	CGT​GAC​GAT​CCT​GAA​GAC​TTG	227
RPA	GGG​GAT​ACA​AAC​ATA​AAG​CCC​A	CGA​TAA​CGC​GGC​GGA​CTA​TT	80
RAD52	CCA​GAA​GGT​GTG​CTA​CAT​TGA​G	ACA​GAC​TCC​CAC​GTA​GAA​CTT​G	145
XRCC5	GTG​CGG​TCG​GGG​AAT​AAG​G	GGG​GAT​TCT​ATA​CCA​GGA​ATG​GA	86
RARA	AAG​CCC​GAG​TGC​TCT​GAG​A	TTC​GTA​GTG​TAT​TTG​CCC​AGC	122
RARB	TCC​GAA​AAG​CTC​ACC​AGG​AAA	GGC​CAG​TTC​ACT​GAA​TTT​GTC​C	125
RARG	TGT​CAC​CGC​GAC​AAA​AAC​TGT	CGA​GGG​GAA​AGT​CTC​CTG​A	234
RXRA	ATG​GAC​ACC​AAA​CAT​TTC​CTG​C	GGG​AGC​TGA​TGA​CCG​AGA​AAG	211
RXRB	ACG​GCT​ATG​TGC​AAT​CTG​C	CGG​ATG​GTG​CGT​TTG​AAG​AA	96
RXRG	CCG​GAT​CTC​TGG​TTA​AAC​ACA​TC	GTC​CTT​CCT​TAT​CGT​CCT​CTT​GA	119

### 2.4 Western blot analysis

HaCaT cells were lysed with RIPA lysis buffer (Beyotime, P0013B), and protein concentrations were quantified using the BCA Protein Assay Kit (Beyotime, P0012). 5X SDS loading buffer (Beyotime, P0015L) was then added to the lysates. Proteins were separated by electrophoresis on 4%–20% SurePAGETM gels (GenScript) and transferred to polyvinylidene fluoride membranes (Millipore, IPVH00010) using an eBlot (GenScript) wet transfer system. The membranes were blocked with 5% (v/v) non-fat milk (Beyotime, P0216) for 3 h. All primary antibodies were diluted in QuickBlock™ Western primary antibody dilution buffer (Beyotime, P0256). The membranes were then incubated with primary antibodies at 4°C overnight. Following this, the membranes were incubated with horseradish peroxidase-conjugated secondary antibodies (Jackson Laboratories) for 2 h at room temperature. Secondary antibodies were applied at a dilution of 1:5,000. Membranes were washed three times with TBST, 10 min per wash. Signals were visualized using an ECL chemiluminescence detection system (SuperSignal West Pico PLUS Chemiluminescent Substrate), and immunoblotting images were collected using an eBlot (Genscript). Protein band intensities were analyzed and quantified using ImageJ software for grayscale measurements.

The following antibodies were used: β-Actin (Proteintech, 81115-1-RR), Collagen I (Proteintech, 14695-1-AP), Collagen Type III (Proteintech, 22734-1-AP), γH2A.X (phospho S139) (Abcam, ab81299), HMGB1 (Proteintech, 10829-1-AP), ATM (phospho S1987) (Abcam, ab315019), P53 (phospho Ser15) (Proteintech, 80195-1-RR), P53 (Proteintech, 10442-1-AP), CHK2 (phospho Thr68) (Proteintech, 29012-1-AP), GAPDH (Abcam, ab8245), RAD52 (Proteintech, 28045-1-AP), and BRCA1 (Proteintech, 22362-1-AP). Goat anti-rabbit and anti-mouse HRP-conjugated secondary antibodies (Jackson, 111-035-003 and 115-035-003) were also employed.

### 2.5 Comet assay

The comet assay was conducted using the Comet Assay Kit (C2041S, Beyotime) according to the manufacturer’s instructions. Briefly, cells were harvested and digested, then combined with low-melting point agarose. This cell-agarose mixture was spread onto a microscope slide that had been pre-coated with a thin layer of agarose. Following the solidification of the agarose, the cells were lysed, and electrophoresis was performed in an alkaline buffer. After neutralization, DNA was stained with propidium iodide (PI), allowing for visualization of DNA fragments using a fluorescence microscope (DMi8, Leica, Germany). Image analysis, including comet images and tail intensity measurements, was performed using ImageJ software.

### 2.6 RHE culture and treatment

SkinEthic™ Reconstructed Human Epidermis (RHE) was obtained from EPISKIN (France). This *in vitro* model consists of normal human keratinocytes cultured on an inert polycarbonate filter at the air-liquid interface, closely resembling the histological structure of *in vivo* human epidermis. Upon arrival, the epidermis was transferred to six-well culture plates containing maintenance medium (SkinEthic™, 24-SMM-0919) and incubated at 37°C with 5% CO2 for 24 h. Following incubation, the epidermis was irradiated with UVB light at a dose of 120 mJ/cm^2^ while submerged in culture medium. After irradiation, the epidermis was embedded in tissue freezing medium (Leica, 14020108926) and rapidly frozen using liquid nitrogen. Subsequently, the epidermis was sectioned into 10 µm slices using a cryostat (Leica, CM1950).

### 2.7 Immunofluorescence staining

HaCaT cells were treated as previously described, then fixed in 4% (v/v) paraformaldehyde for 10 min. The cells were permeabilized with 0.5% (v/v) Triton X-100 for 10 min and subsequently blocked with 5% (v/v) BSA for 1 h at room temperature. Following three washes with PBS, the cells were incubated overnight at 4°C with the primary antibody γH2A.X (phospho S139) (Abcam, ab81299). Afterward, they were incubated for 1 h at room temperature with an Alexa Fluor 594-conjugated goat anti-rabbit secondary antibody (Life Technologies). Nuclei were counterstained with DAPI (Beyotime, China). Cell images were captured using a fluorescent microscope (DMi8, Leica, Germany).

### 2.8 Cell apoptosis assay

Cells were collected and incubated with an Annexin V-FITC/PI Apoptosis Kit (Elabscience, E-CK-A211) following the manufacturer’s protocol. Briefly, HaCaT cells (5 × 10^5^) were collected, washed with PBS, and resuspended with Annexin V-FITC Reagent and 50 μg/mL Propidium Iodide (PI) Reagent, followed by incubation at room temperature in the dark for 20 min. Cells were then analyzed using a flow cytometer (Beckman CytoFlex, United States).

### 2.9 Cell cycle analysis

Cells were collected and incubated with a Cell Cycle Assay Kit (Red Fluorescence) (Elabscience, E-CK-A351) according to the manufacturer’s protocol. Briefly, cells (5 × 10^5^) were collected and washed with PBS, then fixed and permeabilized with 70% (v/v) ethanol at −20°C overnight. Fixed cells were washed with PBS at room temperature for 15 min, resuspended with RNase A reagent at 37°C for 30 min, and stained with 50 μg/mL PI staining solution, followed by incubation at 4°C in the dark for 30 min. Cells were analyzed using a flow cytometer (Beckman CytoFlex, United States), and results were evaluated using ModFit LT (Cytonome Verity, United States).

### 2.10 Bulk RNA sequencing and data analysis

Total RNA was extracted using TRIzol™ Reagent (Gibco, Cat. No. 15596026CN) according to the manufacturer’s protocol. Base calling and preliminary quality analysis of the raw RNA sequencing data were performed using Bcl2fastq (v2.20.0.422), yielding pass-filter data. The quality of the sequencing data was assessed with FastQC (v0.10.1) and filtered using Cutadapt (v1.9.1). Filtered sequencing data were aligned to the reference genome with Hisat2 (v2.2.1). RNA sequencing was conducted on an Illumina NovaSeq™ 6000 platform. Gene abundance was quantified using FPKM (fragments per kilobase of exon model per million mapped reads). For differential gene expression analysis, a false discovery rate (FDR) threshold of <0.05 and fold change criteria of ≥1.5 or ≤0.66 were applied. GOSeq (v1.34.1) was utilized to identify Gene Ontology (GO) terms associated with a list of enriched genes. TopGO (v2.18.0) was employed to visualize the directed acyclic graph (DAG). In-house scripts were used to enrich significant differentially expressed genes in KEGG (Kyoto Encyclopedia of Genes and Genomes) pathways.

### 2.11 Statistical analysis

Data are presented as means ± standard error of the mean (SEM) and were analyzed using GraphPad Prism (version 9.0; GraphPad Software Inc.). A one-way analysis of variance (ANOVA) was performed to assess statistical differences among experimental groups. Tukey’s *post hoc* tests were employed to correct for multiple comparisons. Significant probability values are denoted as *p < 0.05, **p < 0.01, ***p < 0.001, and ****p < 0.0001.

## 3 Result

### 3.1 Identifying the synergistic biological functions of ROL and RPalm following UVB exposure in HaCaT

**FIGURE 1:**
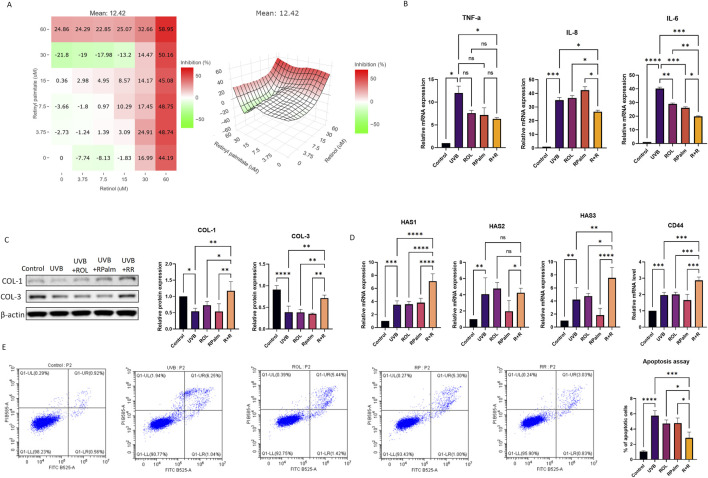
Identifying the Synergistic Biological Functions of ROL and RPalm Following UVB Exposure in HaCaT. **(A)** Dose-response map presented as a heatmap and interactive 3D surface, illustrating the synergistic effect of ROL and RPlam at different dosages. Figure generated by SynergyFinder+. **(B)** Relative mRNA expression levels of TNF-α, IL-6, and IL-8 in HaCaT cells. These cells were either non-UVB treated or UVB treated and subjected to different treatments: ROL, RPlam, ROL + RPlam, or no treatment. **(C)** Western blot analysis and relative protein expression levels of COL-1 and COL-3 in HaCaT cells. These cells were either non-UVB treated or UVB treated and received the same treatments. **(D)** Relative mRNA expression levels of HAS1, HAS2, HAS3, and CD44 in HaCaT cells. These cells were either non-UVB treated or UVB treated and subjected to the same treatments. **(E)** Apoptosis assay of HaCaT cells. The cells were either non-UVB treated or UVB treated and received the same treatments. Data are presented as means ± SD (N = 4). *p < 0.05, **p < 0.01, ***p < 0.001.

To determine the optimal ratio of (ROL) and (RPalm), HaCaT cells were treated with varying concentrations of ROL and RPalm (R+R), ranging from 0 to 60 µM. Using SynergyFinder+, we created a dose-response map, presented as a heatmap and interactive 3D surface, to illustrate the synergistic effects of ROL and RPalm at different dosages (Figure 1A; Zheng et al., 2022). The optimal combination was identified as 15 µM ROL and 30 μM RPalm, which maximized HaCaT cell viability. Subsequent experiments were conducted using this concentration.

Next, we explored the synergistic biological functions of ROL and RPalm. We selected 50 mJ of UVB as the effective dose to induce photodamage (Supplementary Figure 1A). The solvent 0.1% DMSO posed no change in the cell viability before or after the UVB exposure (Supplementary Figure 1B). We first assessed their anti-inflammatory effects following 50 mJ of UVB irradiation. ROL and RPalm were applied to HaCaT after the UVB exposure to avoid the photodegradable effects. As shown in Figure 1B, the combination of ROL and RPalm significantly reduced the UVB-induced mRNA expression of IL-6, and IL-8 in HaCaT cells compared to treatments with ROL or RPalm alone. However, while ROL and RPalm treatments reduced TNF-α mRNA expression upon UVB exposure, there was no significant difference between the effects of ROL alone and RPalm alone. Regarding collagen production, the combination also notably increased the protein expression of COL-1 and COL-3 after UVB exposure (Figure 1C). Furthermore, ROL and RPalm effectively stimulated the gene expression of hyaluronic acid synthases HAS1 and HAS3, as well as CD44, the primary receptor for hyaluronan on the surface of human keratinocytes (Figure 1D) compared to single use of ROL, RPalm and UVB treatment. However, the combination of ROL and RPalm could not increase HAS2 expression compared to UVB and ROL treatment.

Finally, we evaluated the combined effects of ROL and RPalm in reducing UVB-induced cell apoptosis using flow cytometry. As illustrated in Figure 1E, the combination significantly mitigated UVB-induced apoptosis compared to the individual treatments of ROL or RPalm.

### 3.2 Bulk mRNA sequencing of the effects of ROL and RPalm combination on UVB-irradiated HaCaT

**FIGURE 2:**
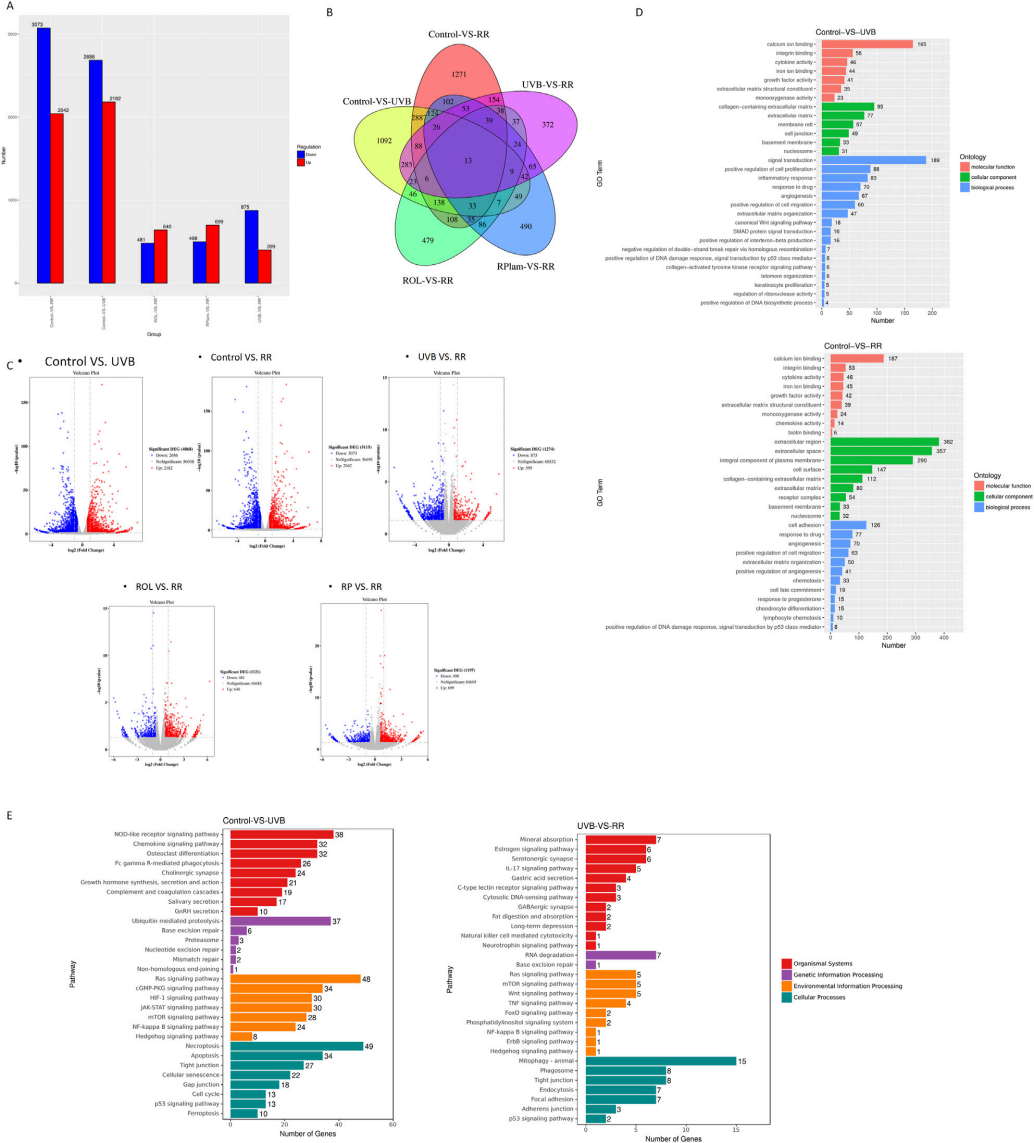
Bulk mRNA sequencing of the effects of ROL and RPalm combination on UVB-irradiated HaCaT. **(A)** The bar chats present the DEG of different treatment groups. **(B)** Venn diagram shows the overlap DEG of different treatment groups. **(C)** Volcano plots of DEG in different treatment groups. Significantly upregulated and downregulated genes are highlighted in red and blue, respectively. **(D)** GO enrichment analysis of DEGs between control HaCaT cells and UVB-irradiated HaCaT cells; control HaCaT cells and R+R-treated UVB-irradiated HaCaT cells. **(E)** KEGG enrichment histogram of DEG for control HaCaT cells and UVB-irradiated HaCaT cells; UVB-irradiated HaCaT cells and R+R-treated UVB-irradiated HaCaT cells.

Bulk mRNA sequencing was conducted on several groups: UVB-exposed HaCaT cells, UVB-exposed HaCaT cells treated with ROL, UVB-exposed HaCaT cells treated with RPalm, UVB-exposed HaCaT cells treated with R+R, and control HaCaT cells (N = 3). As shown in Figures 2A–C, UVB exposure led to the identification of 4,872 differentially expressed genes (DEGs) compared to the control group. The treatment with UVB+R+R resulted in approximately 5,115 DEGs relative to the control, with 1,274 DEGs identified between the UVB-exposed group and the UVB+R+R group.

Gene ontology (GO) analysis revealed significant enrichment of DEGs related to the positive regulation of DNA damage response and signal transduction mediated by p53 in both the control and UVB+R+R groups (Figure 2D). Additionally, KEGG pathway enrichment analysis highlighted the p53 signaling pathway and various genetic information processing pathways associated with DNA damage repair in comparisons between the control and UVB-exposed groups, as well as between the UVB-exposed and UVB+R+R treatment groups (Figure 2E).

### 3.3 Anti-DNA damage effects for ROL and RPalm combination in UVB-exposed HaCaT

**FIGURE 3:**
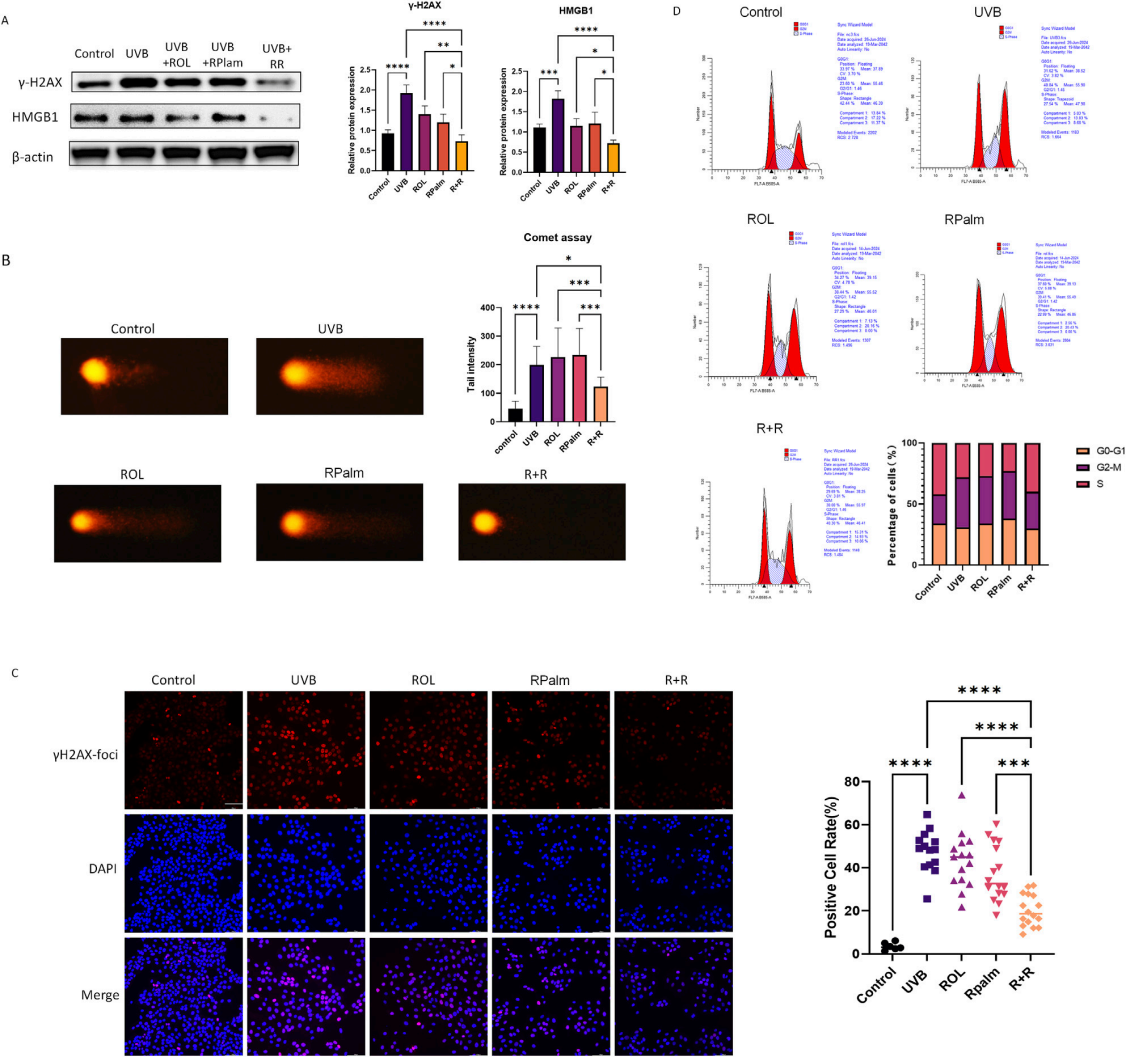
Anti-DNA damage effects for ROL and RPalm combination in UVB-exposed HaCaT. **(A)** Western blot analysis and relative protein expression levels of γ-H2AX and HMGB1 in HaCaT cells. These cells were either non-UVB treated or UVB treated and received different treatments: ROL, RPlam, ROL + RPlam, or no treatment. **(B)** Comet assay showing tail intensity of HaCaT cells. The cells were either non-UVB treated or UVB treated and subjected to the same treatments. Scale bar: 40 µm. **(C)** Immunofluorescence images of γH2AX foci in HaCaT cells. These cells were either non-UVB treated or UVB treated and received the same treatments. Scale bar: 100 µm. **(D)** Cell cycle analysis by flow cytometry for HaCaT cells under five different treatments. The cells were either non-UVB treated or UVB treated and received the same treatments. Data are presented as means ± SD (N = 4). *p < 0.05, **p < 0.01, ***p < 0.001.

UV radiation can induce double-strand breaks (DSB) in DNA. Given our previous findings that the combination of R+R is linked to DNA damage response, we investigated its effects on modulating this response following UVB exposure. As shown in Figure 3A, UVB exposure increased the protein expression of DNA damage markers γ-H2AX and HMGB1 in HaCaT cells. Notably, R+R treatment significantly reduced the levels of these markers compared to treatments with ROL or RPalm alone.

We also performed a comet assay, a sensitive technique for detecting DNA damage at the individual cell level, to evaluate the impact of R+R on DNA integrity in UVB-irradiated HaCaT cells. The results showed that R+R treatment significantly reduced tail intensity after UVB exposure, while neither ROL nor RPalm demonstrated this effect (Figure 3B). To further visualize DSB levels in cells, we conducted immunofluorescence staining for γ-H2AX foci. ROL and RPalm alone did not reduce foci formation upon UVB exposure, whereas R+R treatment effectively decreased foci formation, as illustrated in representative images (Figure 3C). We conducted cell cycle analysis to examine how different treatments affected cell cycle distribution. Our results indicated that UVB irradiation caused an arrest at the G2/M checkpoint, suggesting potential genetic toxicity. In contrast, treatment with R+R shifted the cell cycle into the S phase, indicating enhanced repair of DSBs (Figure 3D).

### 3.4 Synergistic effects of ROL and RPalm in promoting the UVB-induced DNA damage repair in HaCaT

**FIGURE 4:**
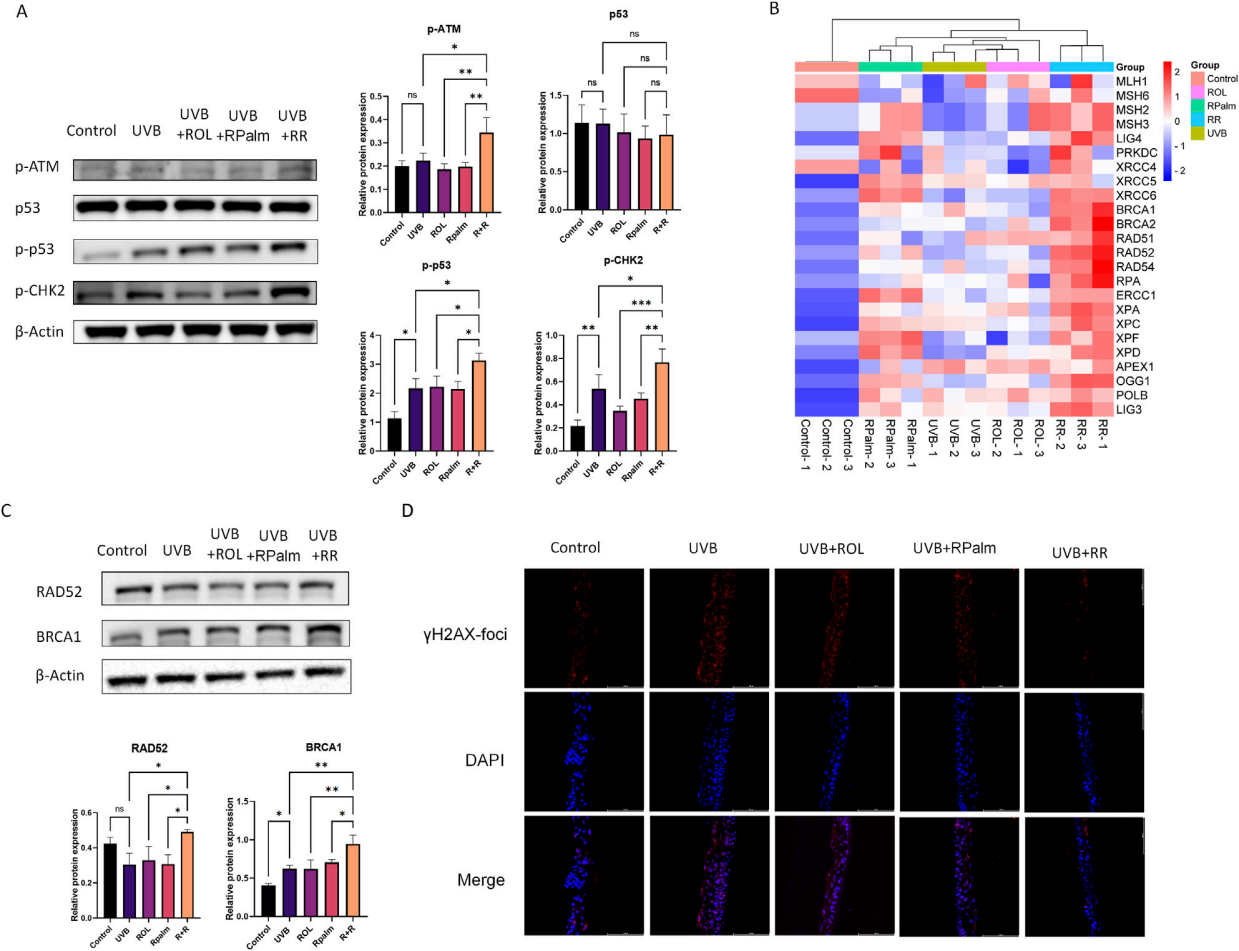
Synergistic Effects of ROL and RPalm in promoting the UVB-Induced DNA Damage Repair in HaCaT. **(A)** Relative protein expression levels of DNA damage response pathway proteins: phosphorylated ATM, p53, phosphorylated p53, and phosphorylated CHK2 in HaCaT cells. These cells were either non-UVB treated or UVB treated and received different treatments: ROL, RPlam, ROL + RPlam, or no treatment. **(B)** Relative mRNA expression levels of DNA damage repair genes presented in a heatmap generated by SRplot. These cells were also either non-UVB treated or UVB treated and subjected to the same treatments. **(C)** Relative protein expression levels of RAD52 and BRCA1 in HaCaT cells, with the same treatment conditions as above. **(D)** Immunofluorescence staining for γH2AX foci in RHE, with the same treatment conditions as above. Scale bar: 100 µm. Data are presented as means ± SD (N = 4). *p < 0.05, **p < 0.01, ***p < 0.001.

Upon UV irradiation, cells activate a DNA damage response (DDR) as a protective mechanism to signal and repair damage. Specifically, the serine-protein kinase ataxia telangiectasia mutated (ATM) plays a crucial role in the DDR, particularly in response to DSB (Kciuk et al., 2020). Therefore, we first examined changes in the ATM-related downstream pathways following UVB exposure and the effects of the R+R treatment in HaCaT cells. As shown in Figure 4A, Western blot analysis revealed that phosphorylated ATM levels were significantly increased in the R+R treatment group compared to the UVB, ROL or RPalm-only group. Additionally, phosphorylated CHK2 and p53(Ser15) were also upregulated in the R+R treatment, indicating activation of the ATM-mediated DDR pathways in response to UVB-induced DNA damage. To identify the specific DNA damage repair mechanisms activated by R+R, we conducted RT-qPCR to assess the mRNA levels of genes associated with five distinct DNA damage repair pathways: nucleotide excision repair (NER), base excision repair (BER), homologous recombination (HR), non-homologous end joining (NHEJ), and mismatch repair (MMR) (Supplementary Figure 2). The heatmap summarizing the RT-qPCR result generated by SRplot in Figure 4B demonstrates that genes related to the HR pathway, including BRCA1 and RAD52 were significantly upregulated in the R+R treatment, as indicated by the deep red coloration (Tang et al., 2023). To validate these findings, we selected BRCA1 and RAD52 for further Western blot analysis. As shown in Figure 4C, the protein expression levels of both BRCA1 and RAD52 were significantly elevated in the R+R group compared to the UVB-only group and groups treated with either ROL or RPalm alone. Finally, we verified the induction of DNA damage and the repair in reconstructed human epidermis (RHE) model. From Figure 4D, we observed that the UVB irradiated RHE significantly upregulated the expression of γH2AX-foci, and the treatment with neither ROL nor RPalm would restore the foci level. However, the application of R+R would reverse the γH2AX-foci expression after the UVB treatment. Collectively, these results suggest that the R+R combination primarily promotes DNA damage repair via the homologous recombination pathway.

### 3.5 The molecular mechanism of ROL and RPalm synergism via RAR in regulating the DNA damage repair

**FIGURE 5:**
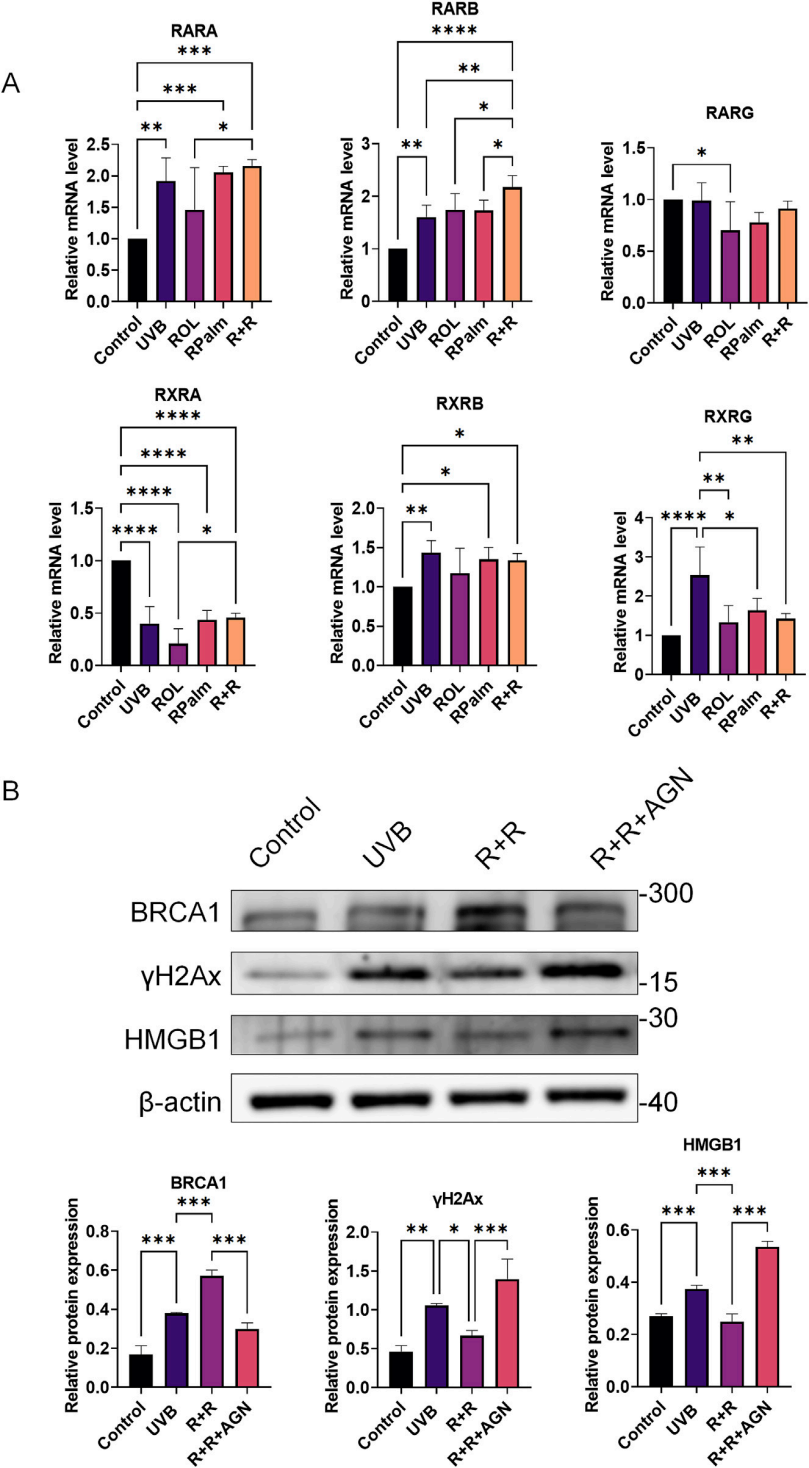
The molecular mechanism of ROL and RPalm synergism via RAR in regulating the DNA damage repair. **(A)** Relative mRNA expression levels of RARα, RARβ, RARγ, RXRα, RXRβ, and RXRγ in HaCaT cells, either treated with UVB or not, and subjected to various treatments: ROL, RPlam, ROL + RPlam, or no treatment. **(B)** Western blot analysis and relative protein expression of BRCA1, γH2AX, and HMGB1 in HaCaT cells, with conditions of non-UVB treatment or UVB treatment, and subjected to treatments: ROL + RPlam, ROL + RPlam with AGN, or no treatment. Data are presented as means ± SD (N = 4). *p < 0.05, **p < 0.01, ***p < 0.001.

To elucidate the underlying molecular mechanisms driving the synergism between ROL and RPalm in regulating DNA damage repair, we examined the retinoic acid receptors (RARs) and retinoid X receptors (RXRs), which are nuclear receptors involved in the regulation of gene expression in response to retinoids (Huang et al., 2014). We screened three main subtypes of RAR: RARα, RARβ, and RARγ, as well as three subtypes of RXR: RXRα, RXRβ, and RXRγ, following the addition of both ROL and RPalm. As shown in Figure 5A, the mRNA expression of RARβ was significantly upregulated in the R+R treatment group compared to the other groups. In contrast, RXRα, and RXRγ and RXRs expression showed no change across the synergistic treatment, indicating that the effects of R+R primarily involved the RAR pathway, likely RXRβ, rather than the RXR pathway. Therefore, we subsequently applied the RAR antagonist AGN193109 (MedChemExpress, United States) to determine whether the DNA repair effect of R+R would be abolished. As indicated in Figure 5B, the addition of AGN193109 significantly dampened the R+R-induced DNA damage repair by decreasing the expression of BRCA1. Moreover, the DNA damage markers γH2AX and HMGB1 were further elevated after the addition of AGN193109 in the R+R treatment, indicating that R+R-induced DNA damage repair is dependent on RAR signaling.

## 4 Discussion

UV radiation has long been recognized as a major trigger in addressing the extrinsic skin photoaging and photodamage. UVB, especially, provides more prominent effects towards the epidermis. Direct photoinduced oxidative stress, most likely ⋅OH, are generated in UVB-irradiated keratinocytes (Wang and Kochevar, 2005). Moreover, UVB is able to produce DNA lesion in an oxygen-independent manner via direct excitation of pyrimidine nucleobases to form CPDs, pyrimidine (6-4) pyrimidone (6-4PPs) in human keratinocytes, resulting deleterious genotoxic effects (Cadet et al., 2015). Increasing evidence suggests that the generation of CPDs, 6-4 PP and DSB upon the UVB irradiation become susceptibility factor for sunlight-induced skin cancer and melanoma in the general population (Perez et al., 2021; Yang et al., 2019). Therefore, there is an urge to find potent ingredients to counteract the UVB induced DNA damage.

Both ROL and RPalm are effective in combating skin aging, each with distinct advantages. RPalm is more stable and less irritating, while ROL offers higher bioactivity. Especially, RPalm is metabolized to ROL through enzymatic hydrolysis by retinyl ester hydrolases, with subsequent transport and potential re-esterification in the body (Gudas, 2022). Numerous studies have highlighted the anti-aging effects of both compounds (Shu et al., 2023; Quan, 2023). Notably, ROL stimulates the production of Type I collagen by regulating the TGF-β/CTGF pathway and significantly reduces CCN1 levels in both intrinsically aged and photoaged skin *in vivo* (Quan et al., 2011). In terms of topical RPalm applied to UVB photoaged mice, the treatment markedly decreased the production of inflammatory cytokines such as IL-6, IL-1β, and TNF-α (Shu et al., 2023). Based on these findings, we hypothesized that combining these two compounds could leverage their strengths, resulting in a balanced and effective anti-aging treatment. In this study, we aimed to unravel the underlying mechanism of the ROL and RPalm combination in combating UVB-induced photodamage. We identified that the optimal ratio of 15 µM ROL and 30 µM RPalm most effectively promoted HaCaT cell proliferation. Additionally, we applied the Combination Subthresholding method to identify the synergistic effects of R+R which were prominent in reducing inflammation and enhancing collagen and hyaluronan synthesis (Duarte and Vale, 2022). These findings align with previous research on the synergistic effects of retinol and its derivatives, where retinol and HRP were reported to promote collagen expression in HFF-1 (Wang et al., 2023).

To better characterize the anti-photodamage mechanism of R+R in our study, we performed RNAseq to unravel the possible functions related to R+R combinations. Our RNAseq and further experiments verified various pathways associated with DNA damage responses and DNA damage repair. Previous study has correlated the reduction of post-UV DNA repair capacity in aging would contribute to the accumulation of DNA damage and the phenotypes of photoaged skin (Moriwaki and Takahashi, 2008). However, several studies also indicate that retinoids are sensitive to photodegradation and photolysis, resulting in less biological activity (Zhong et al., 2024; Fu et al., 2003a). Li et al. also indicated that retinoic acid (RA) could not enhance removal of UV-damaged DNA or repair when the RA was pre- or co-applied with the UV in human keratinocyte (Li et al., 2000). Similar study conducted by Yan et al. also indicated that UVA illumination in the presence of RPalm would lead to DNA strand cleavage (Yan et al., 2005). However, in our study, we have already considered the potential photoinstability issue. Therefore, we especially incubated the retinoids after the UVB exposure to prevent the light-induced degradation and phototoxicity. Meanwhile, currently there is no study indicate the carcinogenetic effects of topical application of ROL and RPalm (Fu et al., 2003a). Indeed, RPalm and its photodecomposition products were not mutagenic in *Salmonella typhimurium* mutation assays and unable to bind to calf thymus DNA, denying the potential of phototoxicity (Fu et al., 2003b). Moreover, pretinol complex that containing β-carotene and Niacinamide as retinol precursors was shown to associate with DNA base repair (Cohen et al., 2020).

Our findings confirmed that UVB exposure induces DSBs, as evidenced by the visualization of γ-H2AX foci. The application of the R+R combination following irradiation effectively reduced DSB levels compared to treatments with ROL or RPalm alone. Previous studies have indicated that the accumulation of γ-H2AX foci after UVB exposure is associated with replication fork collapse due to unrepaired CPDs, which lead to both single- and double-strand breaks (Yang et al., 2019). To further explore the molecular mechanisms behind R+R’s promotion of DNA damage repair, we focused on the ATM-CHK2-p53 signaling pathway. This pathway plays a crucial role in the cellular response to UV-induced DNA damage, particularly in DNA repair processes and cell cycle regulation. ATM is a kinase activated by DNA damage from UV radiation, which phosphorylates several substrates, including p53 (Oh et al., 2011). Phosphorylation of p53 by ATM on specific serine residues dissociates it from Mdm2, preventing degradation and allowing its accumulation to facilitate DNA repair and apoptosis (Chehab et al., 1999). CHK2 is phosphorylated by ATM in response to DNA damage after UV exposure, activating cell cycle checkpoints and facilitating DNA repair (Helt et al., 2005). Low levels of UV radiation cause temporary activation of p53 (phosphorylation on Ser15 and Ser20), leading to cell cycle arrest to aid DNA repair. In contrast, higher levels of UV radiation result in stronger and prolonged p53 activation (phosphorylation on Ser15, Ser20, and Ser46), culminating in apoptosis (Carvalho et al., 2024). Our results verified that R+R activates the ATM-CHK2-p53 signaling pathway, enhancing the DNA damage response. We also considered downstream repair mechanisms in modulating DNA damage. Although UV radiation primarily causes single-strand breaks, the occurrence of DSBs is significant (Strzalka et al., 2020). Eukaryotic cells employ five main DNA repair mechanisms to maintain genomic stability: NER, BER, MMR, NHEJ, and HR. While single-strand breaks are mainly repaired by BER, NER, and MMR, DSBs are predominantly repaired by NHEJ and HR (Shrivastav et al., 2008). We observed that mRNA expression of HR-related genes was significantly upregulated in the R+R treatment groups, indicating the major involvement of this repair pathway. In contrast to NHEJ, HR is considered an error-free repair mechanism, utilizing a homologous sequence, usually the sister chromatid, as a template to ensure high-fidelity repair. The use of the sister chromatid is primarily limited to the S and G2 phases, which correlates with our cell cycle results (Zhang et al., 2009). Initially, DSBs trigger a repair process where the Ku70/80 complex coats the DNA ends. This is then replaced by the MRN complex (MRE11, RAD50, NBS1), which recruits ATM to phosphorylate histone H2AX near the break site (Strzalka et al., 2020). BRCA1 and CtIP are subsequently recruited, along with the MRN complex, to facilitate short resection of the 5′DNA ends, creating a 3′overhang that is coated by RPA. The BRCA1-PALB2-BRCA2 complex then replaces RPA with RAD51, forming a RAD51 filament (Trenner and Sartori, 2019). Repair can proceed through various pathways, including break-induced replication, synthesis-dependent strand annealing, or double Holliday junctions (Heyer et al., 2010). Alternatively, single-strand annealing can occur by end resection factor EXO1. When EXO1 resects the 5′DNA ends, RAD52 facilitates the annealing of complementary single-stranded DNA (Sun et al., 2020). Studies on UVA-induced DSBs have shown that radiation contributes to the formation of the progerin-lamin A complex, which further suppresses 53BP1-mediated NHEJ repair activity (Huang et al., 2017). Moreover, BRCA1 enhances resistance to UV damage, and its interaction with the replication factor C (RFC) at replication forks aids in repair (Pathania et al., 2011). Various natural compounds have been identified to impact DNA repair mechanisms following radiation exposure (Lagunas-Rangel and Bermúdez-Cruz, 2020). For example, curcumin inhibits DNA cross-link damage repair via the Fanconi anemia (FA)/BRCA pathway to sensitize resistant cancer cells (Chen et al., 2015). Despite excision repair, such as NER or BER, is known to direct UV-induced DNA repair, HR-related pathway is also found to eliminate UV-induced CPD damage in various organisms (Eppink et al., 2011; Ries et al., 2000; Sinha and Hader, 2002; McCready et al., 2005). In our study, we highlights the potential of R+R in promoting HR DSB repair through the activation of the ATM-CHK2-p53 signaling pathway, BRCA1 and RAD52.

To elucidate the molecular mechanisms underlying the synergistic effect of R+R in promoting UVB-induced DNA damage repair, we focused on retinoic acid receptors (RARs) and retinoid X receptors (RXRs). RARs α, β, and γ, along with RXRs α, β, and γ, function as ligand-dependent transcription factors activated by retinoids. Heterodimers formed by RARs and RXRs regulate the expression of various genes in the skin and other tissues, with their transcriptional activity dependent on the availability of RAR-activating ligands (Gericke et al., 2013). The RAR-RXR signaling pathways are crucial for immune modulation and skin physiology, influencing skin cell proliferation, differentiation, apoptosis, and epidermal barrier function. Moreover, retinoid metabolism and concentrations in the skin are tightly regulated to maintain adequate levels of the endogenous pan-RAR activator, all-trans retinoic acid (ATRA) (Chapellier et al., 2002). Our findings showed that following UVB exposure, R+R treatment significantly upregulated the mRNA expression of RARs, particularly RARβ. Notably, the application of an RAR antagonist abolished the DNA damage repair promotion by R+R. Maria et al. demonstrated that C286, a RARβ agonist, could enhance DNA repair mechanisms via BRCA1 and ATM to alleviate neuropathic pain following nerve injury (Goncalves et al., 2019). This study underscores the interaction between RAR signaling and DNA repair mechanisms, aligning with our findings.

**FIGURE 6:**
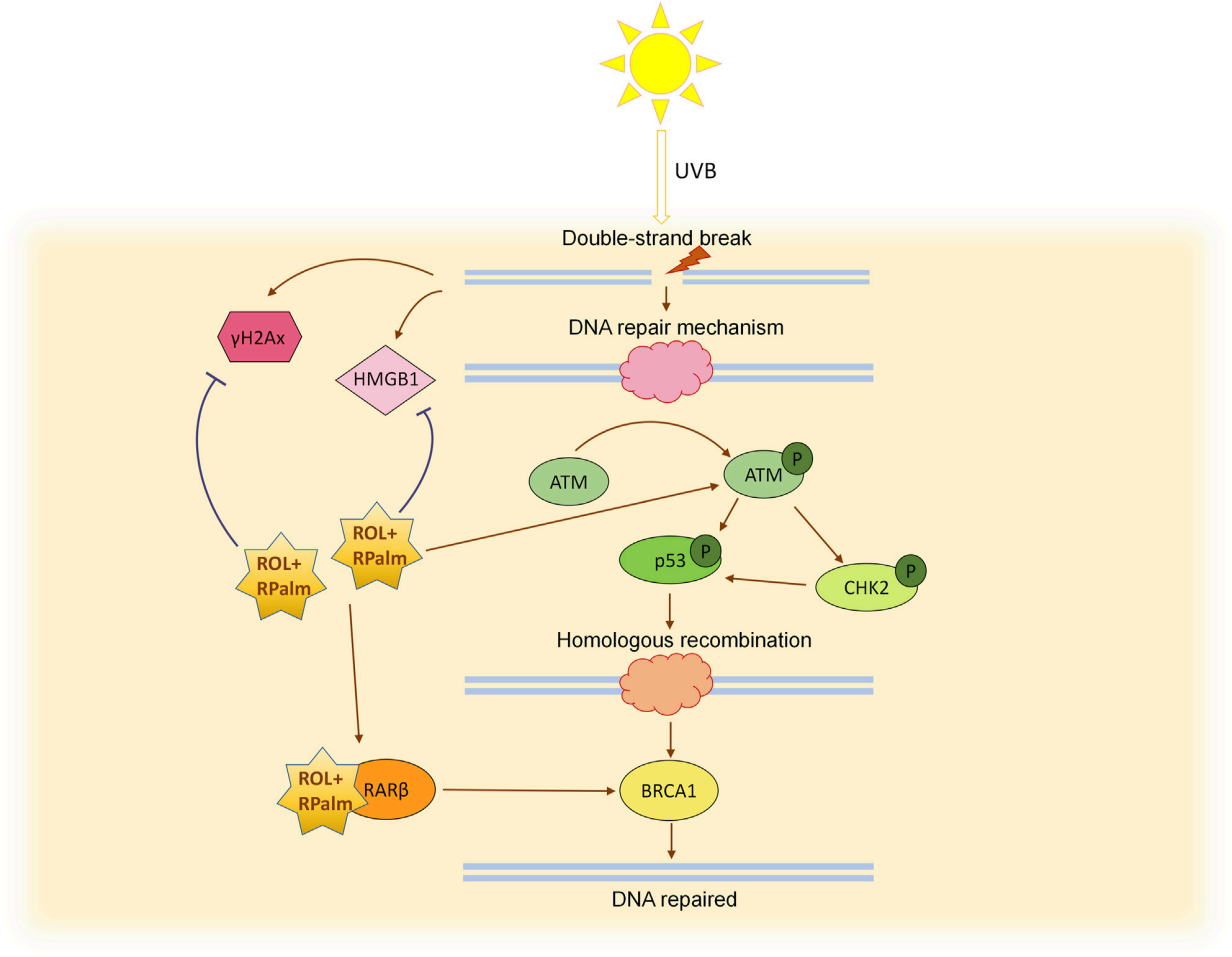
Schematic diagram illustrating the mechanism of ROL + RPlam (R+R) in mitigating UVB-induced DNA damage and promoting homologous recombination repair.

In summary, our research demonstrates that the combined application of ROL and RPalm can enhance the repair of UVB-induced photodamage in human keratinocytes and verified in RHE. This effect is mediated through the ATM-CHK2-p53 signaling pathway and by increasing the expression of HR-associated repair genes via RARβ activation (Figure 6). This study highlights the significant synergistic effects of ROL and RPalm in combating photodamage and photoaging, providing insights for the development of effective cosmetic formulations. It should be noted that this study has examined only in the *in vitro* cellular model and future research would focus on *in vivo* animal studies to validate these findings and confirm the outcomes through clinical trials. We also take into consideration that UVB-induced DSBs are not the only way of damage; incomplete repair of CPDs, 6-4PPs, and replication fork collapse may also cause defects, which should be investigated in future studies. Moreover, from our bulk-seq data, we also found that other relevant pathways, such as NF-κB, JAK-STAT, Hedgehog, and apoptosis, could potentially be related to the synergistic effects of ROL and RPalm in combating photodamage. These data warrant further study to gain a deeper understanding of their synergy effect. Additionally, integrating novel lipid-based nano-vehicle delivery systems could enhance the stability of retinoids. Such advancements may lead to the development of safer and more effective retinoid formulations in the future.

## Data Availability

The datasets presented in this study can be found in online repositories. The names of the repository/repositories and accession number(s) can be found in the article/Supplementary Material.
