# Supplementary figures and images for: Synergistic effects of retinol and retinyl palmitate in alleviating UVB-induced DNA damage and promoting the homologous recombination repair in keratinocytes
Source: Front Pharmacol. 2025 Apr 24;16:1562244. doi: 10.3389/fphar.2025.1562244 (PMC12058701; doi:10.3389/fphar.2025.1562244)

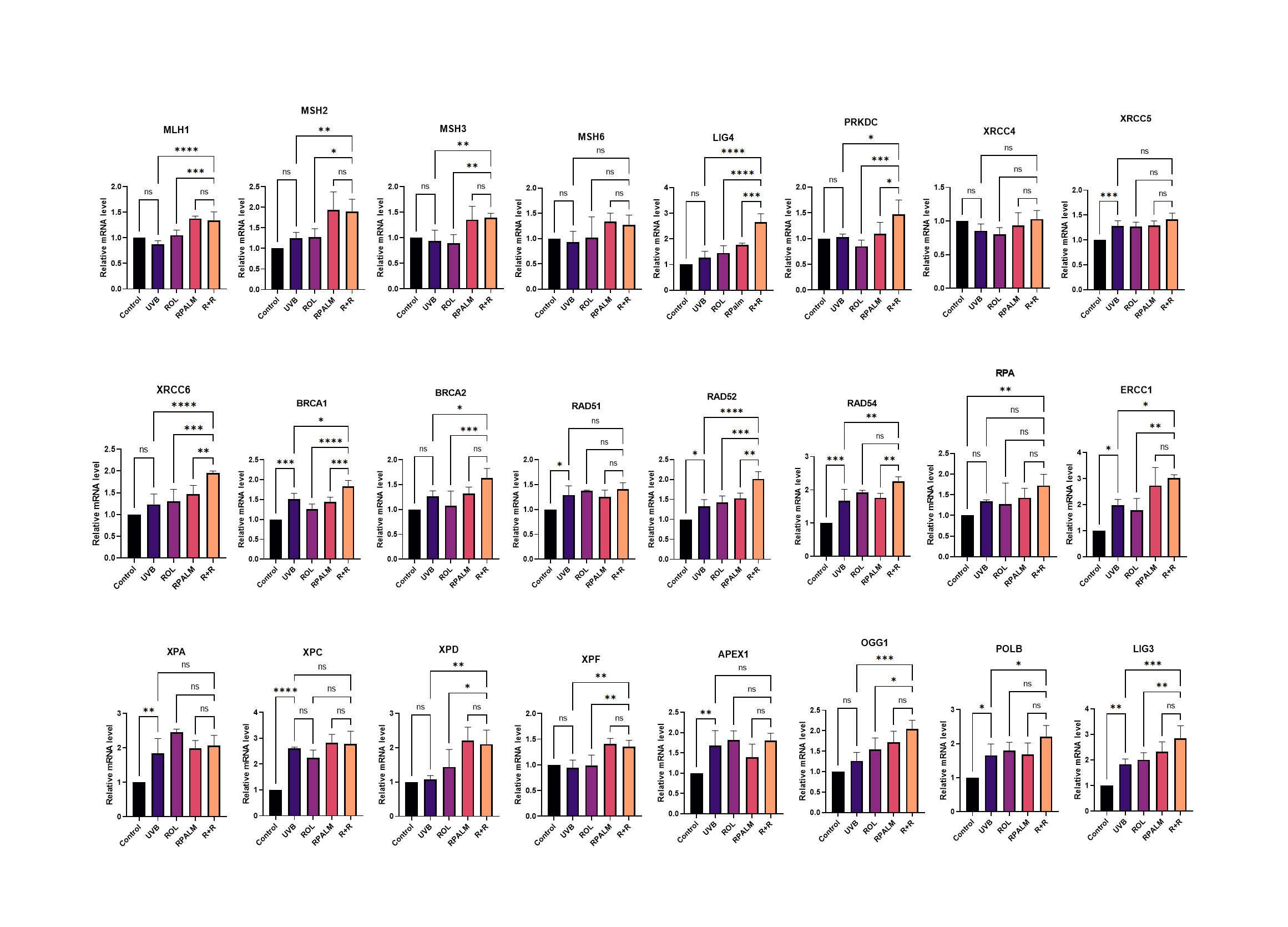

Supplement: Supplementary file 1 [file Image2.tif]

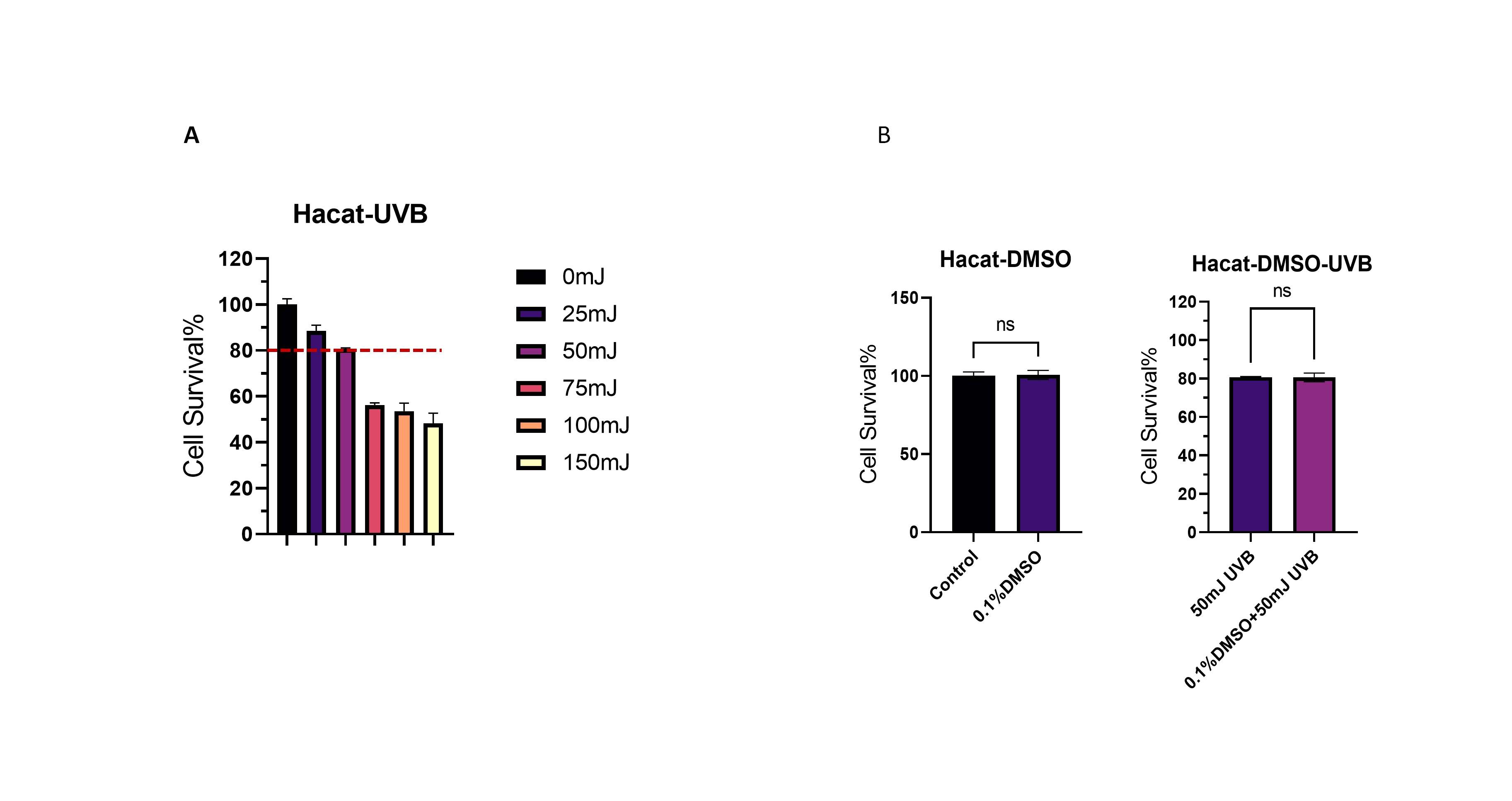

Supplement: Supplementary file 2 [file Image1.tif]
